# Multidirectional Effects of Tormentil Extract on Hemostasis in Experimental Diabetes

**DOI:** 10.3389/fphar.2021.682987

**Published:** 2021-05-05

**Authors:** Natalia Marcinczyk, Agata Gołaszewska, Anna Gromotowicz-Poplawska, Tomasz Misztal, Jakub Strawa, Michał Tomczyk, Irena Kasacka, Ewa Chabielska

**Affiliations:** ^1^ Department of Biopharmacy, Medical University of Bialystok, Bialystok, Poland; ^2^ Department of Physical Chemistry, Medical University of Bialystok, Bialystok, Poland; ^3^ Department of Pharmacognosy, Medical University of Bialystok, Bialystok, Poland; ^4^ Department of Histology and Cytophysiology, Medical University of Bialystok, Bialystok, Poland

**Keywords:** hemostasis, ellagitannins, STZ-induced diabetes, thrombosis, *Potentilla erecta*

## Abstract

In our previous study, we showed that ellagitannin- and procyanidin-rich tormentil extract (TE) decreased experimental arterial thrombosis in normoglycemic rats through platelet inhibition. TE also slightly increased coagulation and attenuated fibrinolysis; however, these effects did not nullify the antithrombotic effect of TE. The present study aimed to assess whether TE exerts antithrombotic activity in streptozotocin (STZ)-induced diabetes, which is characterized by pre-existing increased coagulation and impaired fibrinolysis, in *vivo* and *ex vivo* thrombosis assays. TE (100, 200, or 400 mg/kg, *p. o.*) was administered for 14 days to STZ-induced diabetic rats and mice. TE at 100 mg/kg dose decreased the thrombus area in the mice model of laser-induced thrombosis through its potent antiplatelet effect. However, TE at 200 mg/kg dose increased thrombus weight in electrically induced arterial thrombosis in rats. The prothrombotic effect could be due to increased coagulation and attenuated fibrinolysis. TE at 400 mg/kg dose also improved vascular functions, which was mainly reflected as an increase in the arterial blood flow, bleeding time prolongation, and thickening of the arterial wall. However, TE at 400 mg/kg dose did not exert antithrombotic effect. Summarizing, the present results show that TE may exert multidirectional effects on hemostasis in STZ-induced diabetic rats and mice. TE inhibited platelet activity and improved endothelial functions, but it also showed unfavorable effects by increasing the activity of the coagulation system and by inhibiting fibrinolysis. These contrasting effects could be the reason for model-specific influence of TE on the thrombotic process in STZ-induced diabetes.

## Introduction

The present study is a continuation of our previous work wherein we showed the antithrombotic activity of *Potentilla erecta* rhizome extract in normoglycemic rats and mice (Marcinczyk et al., 2017). The genus *Potentilla* L (Rosaceae family) consists of approximately 700 species of annual and biennial plants and small shrubs. Extract from the underground parts of *Potentilla erecta* (L.) Raeusch (tormentil extract [TE]) has been used in traditional ethnomedicine for the treatment of diarrhea and mild inflammation of the oral mucosa because of its antioxidant and anti-inflammatory activity (Tomczyk and Latté, 2009; Melzig and Böttger, 2020; Augustynowicz et al., 2021). The pharmacological properties of tormentil have been discussed in several reviews and it’s monographs are included in the Russian Pharmacopoeia (14th edition), State Pharmacopoeia of the Republic of Belarus, ESCOP, in the British Pharmacopoeia, or the European Pharmacopoeia (10th edition) (Shikov et al., 2021). These actions are induced by ellagitannins and procyanidins, which are rarely found together in a plant material, although the presence of ellagitannins and procyanidins in TE was confirmed by us and other researchers (Geiger et al., 1994; Mari et al., 2013; Marcinczyk et al., 2017). Ellagitannins and procyanidins are characterized by poor bioavailability after oral intake. However, their gut microbiota-synthesized metabolites have good bioavailability and are responsible for *in vivo* activity of TE. Urolithins are metabolites of ellagitannins (Piwowarski et al., 2017), while phenolic acids as well as dimeric and trimeric procyanidins are metabolites of high-molecular-weight procyanidins (Sano et al., 2003; Sánchez-Patán et al., 2012). It has been shown that procyanidins and ellagitannins exert multidirectional effects on hemostasis; however, most of these studies have been conducted *in vitro*, which makes it difficult to predict the activity of this class of compounds after oral intake. The effect of ellagitannins on platelet activity, coagulation, and fibrinolysis was mainly studied *in vitro* by using plasma (Dong et al., 1998), washed platelets (Shen et al., 2003), and chromogenic substrates (Zhang et al., 2004; Yamamoto et al., 2011). The effects of ellagitannin-rich products other than TE on hemostasis has also been studied in humans after oral intake. The consumption of pomegranate juice has been shown to decrease collagen-induced platelet aggregation (Haber et al., 2011) while blended frozen red raspberries were found to improve flow-mediated vasodilation of the brachial artery (Istas et al., 2018). The effects of procyanidins on hemostasis reported thus far include mainly increase in nitric oxide (NO) and prostacyclin (PGI_2_) production (*in vitro* and *in vivo*) (Schramm et al., 2001; Byun, 2012; Novakovic et al., 2017) and decrease in platelet activity (*in vitro* and *in vivo*) (Murphy et al., 2003; Bouaziz et al., 2007; Morel et al., 2014). Furthermore, by using stasis-induced model of venous thrombosis in rats (Jiang et al., 2007) and laser-induced model of arterial thrombosis in mice, the antithrombotic effect of procyanidins after oral intake was also demonstrated (Sano et al., 2005).

In our previous study, we showed for the first time the antithrombotic activity of ellagitannin- and procyanidin-rich TE in an animal model. In our study, TE inhibited arterial thrombosis in normoglycemic rats through a mechanism dependent on platelet inhibition. The underlying mechanism of this antiplatelet effect was based on the inhibition of thromboxane (TXA_2_) production in platelets, which was comparable to the effect of acetylsalicylic acid. We also showed that TE slightly enhanced fibrin formation and attenuated fibrinolysis, but these effects did not abolish the antithrombotic effect of TE (Marcinczyk et al., 2017).

Considering the multidirectional activity of TE in normoglycemic rats, the main goal of the present study was to assess whether TE exerts the antithrombotic effect in streptozotocin (STZ)-induced diabetes, i.e., in conditions with intrinsically increased fibrin formation and impaired fibrinolysis. Hyperglycemia in type 1 diabetes is a risk factor for cardiovascular diseases and thromboembolic complications (Lee et al., 2019). Enhanced coagulation, impaired fibrinolysis, and increased platelet activity expressed as an enhanced production of TXA_2_ and P-selectin secretion are often observed in patients with diabetes (Wisinski and Kimple, 2016; Sobczak and Stewart, 2019). Diabetes also impairs endothelial functions and vessel wall contractility and leads to a reduction in blood flow (Lunder et al., 2018), which may affect the dynamics of thrombus formation. Furthermore, vascular complications of diabetes, such as macro- and microangiopathy, contribute to hemostasis activation (Tarnow et al., 2009; Zahran et al., 2018). Because of multidirectional hemostasis dysfunctions, the evaluation of the activity of TE, which acts on multiple targets, in diabetes seems to be justified.

In our present study, we used a model of STZ-induced diabetes (STZ-diabetes), which clinically reflects type 1 diabetes (Furman, 2015), and conducted experiments in two models of thrombosis (electrically induced and laser-induced thrombosis) that differ in the mechanism of thrombus formation. Electrically induced arterial thrombosis allows to assess thrombus weight and blood flow disturbances due to vessel-occluding thrombus formation and growth after extensive endothelium damage. Dynamics and the extent of the thrombotic process assessed in this model involve both hemodynamic conditions and hemostatic activity. Thrombus formed in this model is mainly composed of platelets and fibrin, with a minor amount of red and white blood cells (Zakrzeska et al., 2015). The model of laser-induced thrombosis combined with confocal imaging was used for the intravital observation of the thrombotic process at the site of mesenteric vein injury, where thrombus formation occurs on the exposed subendothelial matrix. Because the thrombus is composed of platelets, this model allows to assess the thrombus area and platelet activation (Marcinczyk et al., 2020). Furthermore, by using a wide range of advanced techniques, we determined the effect of TE on the components of hemostasis: platelets, coagulation system, fibrinolysis, and endothelial-dependent vascular functions. This enabled more detailed and multidimensional study of the effect of TE on the thrombotic process and hemostasis in STZ-diabetes.

## Materials and Methods

### Preparation of Tormentil Extract and LC-MS Analysis

The TE extract used in the study is the same as described by Marcinczyk and co-authors (2017). It was prepared in the same extraction protocol, and can clearly correlate these two datasets. The powdered plant material (2.0 g) (batch number: 268.2020420.2020; Kawon, Gostyń, Poland) was extracted with 150 ml of 80% (v/v) methanol in an ultrasonic bath (Sonic-5, Polsonic, Poland) at a controlled temperature (40 ± 2°C) for 45 min. After solvent evaporation under reduced pressure and vacuum controlled temperature (Büchi System, Flawil, Switzerland) (temperature: 40 ± 2°C) the extract was suspended in water and subjected to lyophilization using a vacuum concentrator (Labconco, Kansas City, United States) until a constant weight of the extract was obtained (yield 0.89 g; 44.5%). Details of LC-ESI-MS analysis of the TE extract have been also described previously (Marcinczyk et al., 2017).

### Animals

Male Wistar rats (weighing 260–290 g) and male C57BL6 mice (weighing 24–29 g) were used in the study. Experiments were conducted in accordance with EU Guidelines on Animal Experiments (European Directive 2010/63/EU). All procedures involving animals and their care were approved by the Local Ethical Committee on Animal Testing (Approval Nos.: 72/2018 and 73/2018).

Before conducting procedures of arterial thrombosis, laser-induced thrombosis, and assessment of P-selectin secretion and tissue factor (TF) expression, rats and mice were anesthetized. Rats were anesthetized with a single intraperitoneal injection of pentobarbital (40 mg/kg, *i. p.*, Morbital, Poland). Mice were anesthetized with a single injection of ketamine and xylazine mixture (120 mg/kg, *i. p*., Ketamina 10%, Biowet, Poland; 12.5 mg/kg, *i. p.*, Xylapan, Biowet, Poland). After the experiments, mice were euthanized by cervical dislocation, while rats were anesthetized with pentobarbital overdose (200 mg/kg, *i. p.*).

### Diabetes Induction

Diabetes was induced in rats by a single intraperitoneal injection of STZ (Sigma Aldrich, Steinheim, Germany) at the dose of 65 mg/kg. Rats from the control group (rats without diabetes, referred as VEH) were injected with an equal volume of a citrate buffer. Blood glucose level was measured on the third day after STZ injection by using an Optium Xido glucometer (Abbott, United States). Diabetes was defined as a blood glucose level of >200 mg/dl. The development of diabetes in rats occurred over a period of 5 weeks. The blood glucose level was again measured 5 weeks after STZ injection.

Diabetes was induced in mice by a single intraperitoneal injection of STZ at 200 mg/kg dose. Mice from the control group (VEH) were injected with an equal volume of a citrate buffer. The blood glucose level was measured on the third day after STZ injection. Diabetes was defined as a blood glucose level of >200 mg/dl. The blood glucose level was again measured 4 weeks after STZ injection.

### TE Administration

Diabetic mice and rats received TE *per os* (*p.o.*) with an oral gastric tube, twice daily at doses of 100, 200, and 400 mg/kg in a volume of 3 ml/kg in 5% water solution of gum arabic. Rats and mice from the VEH groups (normoglycemic rats and mice) and the Diabetes groups (rats and mice with diabetes that did not receive TE) received an equal volume of a 5% water solution of gum arabic.

### Primary Hemostasis Template Bleeding Time (BT) in Rats (*in vivo*)

Template bleeding time was measured according to Dejana et al. (Dejana et al., 1982) before the procedure of arterial thrombosis induction.

### Electrically Induced Arterial Thrombosis in a Rat Carotid Artery (*in vivo*)

Induction of arterial thrombosis (Schumacher et al., 1993) was performed according to our modified method (Marcinczyk et al., 2017). Arterial thrombus formation was induced by electrical stimulation (1 mA, 10 min) of the right common carotid artery. Thrombus progression led to a gradual reduction in the carotid blood flow, which was monitored with a Doppler flow probe (Transonic Systems Inc., Ithaca, United States) connected to a blood flowmeter (HSE-TRANSONIC Transit Time Flowmeter, Germany). Initial blood flow (IBF) and total time to occlusion (TTO), which was defined as the time from the commencement of the stimulation to the lack of arterial blood flow due to artery occlusion by the thrombus, were measured. Fifty-five minutes after the commencement of electrical stimulation, the thrombus was removed, dried at room temperature, and weighed after 24 h. For *ex vivo* experiments, blood samples were collected from the right heart ventricle by using 3.13% sodium citrate solution (1:10, *v*/*v*) as an anticoagulant.

### Histological Staining of Rat Arterial Thrombus

Fragments of the carotid artery with thrombus were acquired from three rats of the following groups: VEH, Diabetes, 100 mg/kg, 200 mg/kg, and 400 mg/kg. These fragments were immediately fixed in 10% buffered formalin and routinely embedded in paraffin. Thrombus paraffin blocks were cut into section of 4 µm thick, then stained with hematoxylin-eosin for general histological examination. An experienced histologist, blinded to the technique used, assessed the slides in terms of their overall histological quality. Histological preparations were evaluated using an Olympus BX43 light microscope (Olympus 114 Corp., Tokyo, Japan) with an Olympus DP12 digital camera (Olympus 114 Corp., Tokyo, Japan) and documented.

### Confocal Microscopy Observation

In the experiments of laser-induced thrombosis, P-selectin secretion, TF expression, and visualization of fibrin net and thrombus formation on the collagen, a fixed-stage microscope Zeiss Axio Examiner Z1 (Carl Zeiss Microscopy GmbH, Germany), a confocal scanner unit (CSU-X1, Yokogawa Electric Corporation, Japan), and a W Plan-Apochromat 20×/1.0 water immersion objective (Carl Zeiss Microscopy GmbH) were used. SlideBook 6.0 (Intelligent Imaging Innovations, Inc., United States) was used to analyze the recordings and images.

(1) Laser-induced thrombosis in the mice mesenteric vein and the assessment of thrombus area and PECAM-1/thrombus ratio (*in vivo*)

Laser-induced thrombosis was performed to establish the influence of TE on thrombus formation and to assess the activity of thrombus-forming platelets. Laser-induced thrombosis was performed as described previously (Marcinczyk et al., 2020). Briefly, 5 min before mesentery vein wall damage, Alexa Fluor 647-labeled PECAM-1 antibody (Alexa Fluor 647 anti-mouse CD31 antibody, BioLegend, United States) was injected into the femoral vein. To visualize the vessel wall and phosphatidylserine (PS)-negative platelets, 3,3′-dihexyloxacarbocyanine iodide (DiOC_6_(3), 0.1 mM in 0.05 ml of the mixture of DMSO and PBS (volume ratio 1:50); Life Technologies, Molecular Probes, United States) was administered by an intramuscular injection 5 min before thrombosis induction. A midline laparotomy incision was then made, and the mesentery of the ileum was then pulled out of the abdomen and draped over a plastic mound. The mesentery vein was examined microscopically and identified. The mesentery was continuously perfused with prewarmed (37°C) PBS to prevent the vessels from drying. The mesentery vein wall was injured by a 532 nm argon ion ablation laser (Ablate™, Intelligent Imaging Innovations, Inc., United States). The induction and progression of thrombosis were recorded for 3 min. One record was divided into 25 time points. In each time point, the area of thrombus was encircled. The values of the thrombus area from each time point were added and referred to as the total thrombus area. To assess the activity of platelets in thrombus, the area of fluorescence of PECAM-1 was also measured at each time point. The area of PECAM-1 fluorescence at a particular time point was then divided by the thrombus area at that time point. The values from one record were added and referred to as the PECAM-1/thrombus ratio. One thrombus was induced in one mouse.

(2) P-selectin secretion at the site of laser injury in the mesenteric vein in mice (*in vivo*)

Five minutes before mesentery vein wall damage, Alexa Fluor 647-labeled P-selectin antibody (50 μg/kg, Alexa Fluor 647 Rat Anti-Mouse CD62 P, BD Pharmingen, United States) was injected into the femoral vein of anesthetized mice. The mesenteric vein was isolated and injured by the laser as described above. P-selectin secretion was recorded for 6 min. The record was then divided into 25 time points. The values of P-selectin fluorescence from the 25 time points were added and referred to as total P-selectin fluorescence. One P-selectin measurement was performed in one mouse.

(3) TF expression at the site of laser injury in the mesenteric artery in mice (*in vivo*)

Five minutes before mesentery arterial wall damage, Alexa Fluor 488-labeled TF antibody (35 μg/kg, CD142 Antibody, Alexa Fluor 488 conjugated, Bioss Inc., United States) was injected into the femoral vein of anesthetized mice. The mesenteric artery was isolated and injured in the same manner as that for the mesenteric vein. TF expression was recorded for 6 min. One record was divided into 25 time points. The value of TF fluorescence from each time point was multiplied by the area of its fluorescence. The resultant values from 25 time points were added and referred to as total TF fluorescence. One measurement of TF expression was performed in one mouse.

(4) Thrombus formation in a flow chamber and the assessment of platelet procoagulant index (PI) in rat blood (*ex vivo*)

Thrombus formation on collagen was performed to assess the procoagulant activity of platelets. The procoagulant index indicates the amount of irreversibly activated platelets with exposed PS which catalyzes the coagulation reaction. Thrombus formation on collagen type I fibers has been reported earlier (Misztal et al., 2019). Briefly, blood treated with the anticoagulant trisodium citrate was supplemented with Fraxiparine (5 U/mL, GlaxoSmithKline, United Kingdom) and incubated with DiOC_6_(3) (0.1 mM in 0.05 ml of a mixture of DMSO and PBS (volume ratio 1:50)), Life Technologies, Molecular Probes, United States) for 2 min and then supplemented with MgCl_2_ and CaCl_2_ (final concentration of both: 3 mM). For thrombus formation, blood was perfused through a chamber with collagen-coated surface for 4 min at the shear rate of 1000 s^−1^. The shear rate reflected arterial circulation. The thrombus-coated area was then perfused for 3 min with HEPES buffer supplemented with Alexa Fluor 647-conjugated Annexin V (5 μg/ml) (ANX V, Alexa Fluor® 647 conjugate, Thermo Fisher Scientific, United States), which stains PS-positive platelets, and CaCl_2_ (2 mM). The staining process was followed by washing with HEPES buffer without CaCl_2_ and ANX V. Platelets that did not undergo irreversible activation (PS-negative platelets, aggregating platelets) were stained with DiOC_6_(3), which is a lipophilic dye that penetrates through the intact cell membrane. End-stage measurements for thrombus formation were performed by acquiring two-color images of thrombus composed of PS-positive platelets (labeled with ANX V) and PS-negative platelets (visualized with DiOC_6_(3)). To determine the PI, the area of PS-positive platelets was divided by the area of PS-negative platelets.

(5) Evaluation of fibrin net density in rat plasma (*ex vivo*)

Fibrin net density in clot was evaluated as described previously (Gromotowicz-Poplawska et al., 2019). Briefly, rat blood samples were centrifuged to obtain platelet-rich plasma (PRP, 200 × *g* for 20 min) and platelet-poor plasma (PPP, centrifugation of PRP at 14,000 × *g* for 5 min). Alexa Fluor 488-labeled human fibrinogen (Fibrinogen from Human Plasma, Alexa Fluor™ 488 Conjugate, Thermo Fisher Scientific, United States; final concentration: 15 µM) was added to the samples of PRP and PPP. To induce clot formation, CaCl_2_ (final concentration: 20 mM) was added. The samples were then incubated at 37°C for 2 h. Relative clot density was established from the images of the resultant clots. In each image, five 40 µm long straight lines were placed randomly. The number of fibrin fibers crossing each line was counted. The average of the resultant values was then referred to as the relative clot density.

### Biochemical Analysis of Hemostasis in Rat Plasma (*ex vivo*)

Nitrite and nitrate (NO_2_^−^ and NO_3_^−^) concentrations in rat plasma were determined by an assay using the Griess method (R and D Systems, United States). Rat plasma concentrations of active form of tissue plasminogen activator (t-PA), active form of plasminogen activator inhibitor 1 (PAI-1), plasminogen (Innovative Research, Inc., United States), 6-keto prostaglandin 1 α (6-keto PGF_1α_, stable metabolite of PGI_2_, Cayman Chemicals, United States), and tumor necrosis factor-α (TNF-α, Abcam, United Kingdom) were measured by immunoenzymatic assays. The microplate reader ELx808 (BioTek Instruments, Inc., United States) was used in all assays.

### Euglobulin Clot Lysis Time (ECLT) in Rat Plasma (*ex vivo*)

The euglobulin clot lysis time was used to determine the time of clot dissolution. The ECLT was measured according to the method of Tomczyk et al. (Tomczyk et al., 2016).

### Statistical Analysis

Data were evaluated using GraphPad Prism 5. The Shapiro-Wilk test was performed to determine the normal distribution of the data. Differences between two groups were assessed using Student’s *t*-test or paired samples *t*-test (for normally distributed data) or the Mann-Whitney *U* test (for non-normally distributed data). Data are expressed as mean ± SEM or median (interquartile range) of the number of determination (n). A *p* value of <0.05 was considered to be significant.

## Results

### General Characteristic of the Animals

TE did not affect the body weight and blood glucose levels in rats and mice. The mean initial body weight of the rats was 282–293 g. The mean body weight of the diabetic rats (Diabetes, 100 mg/kg, 200 mg/kg, and 400 mg/kg groups) after 5 weeks of experiment was 224–232 g, whereas the mean body weight of rats from the VEH group was 390 g. The mean initial blood glucose level of the rats was 82–85 mg/dl. The mean blood glucose level of the diabetic rats (Diabetes, 100 mg/kg, 200 mg/kg, and 400 mg/kg groups) after 5 weeks of experiment was 374–388 mg/dl, whereas the mean glucose level of rats from the VEH group was 85 mg/dl. The mean initial body weight of the mice was 27–28 g. The mean body weight of the diabetic mice (Diabetes, 100 mg/kg, 200 mg/kg, and 400 mg/kg groups) after 4 weeks of experiment was 21–22 g, whereas the mean body weight of mice from the VEH group was 31 g. The mean initial blood glucose level of the mice was 127–128 mg/dl. The mean blood glucose level of the diabetic mice (Diabetes, 100 mg/kg, 200 mg/kg, and 400 mg/kg groups) after 4 weeks of experiment was 445–460 mg/dl. Based on the daily observation of the animals, no reduced water and food intake, diarrhea or apathy were observed.

### Primary Hemostasis

**FIGURE 1:**
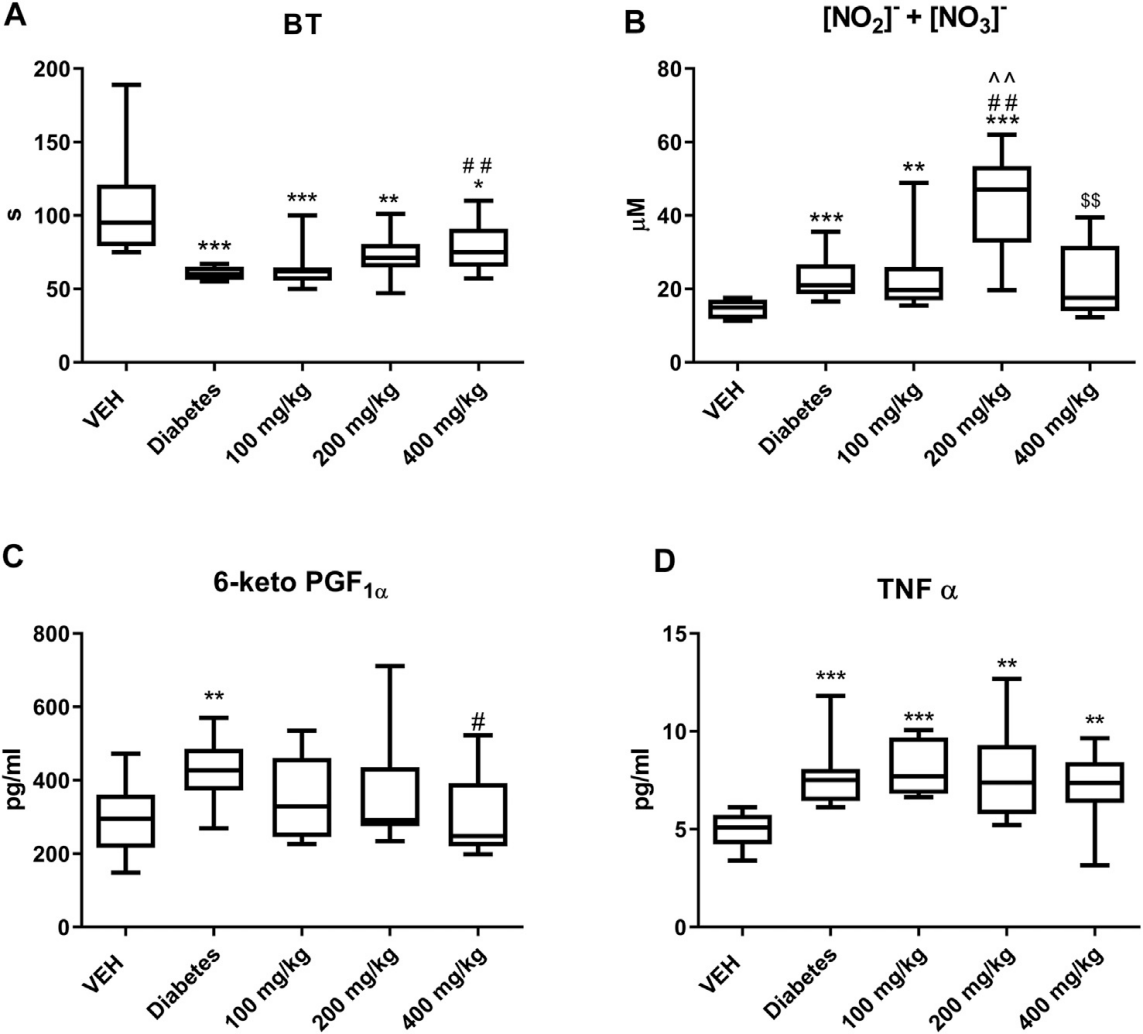
The effect of TE on: BT **(A)**, concentration of NO_2_^−^ and NO_3_^−^ **(B)**, concentration of 6-keto PGF_1α_ **(C)**, concentration of TNF-α **(D)**. *p < 0.05, **p < 0.01, ***p < 0.001 vs. VEH; #*p* < 0.05, ##*p* < 0.01 vs. Diabetes; ^^*p* < 0.01 vs. 100 mg/kg; $$*p* < 0.01 vs. 200 mg/kg; *n* = 8–11. Data are shown as median (interquartile range).

TE prolonged BT only at the dose of 400 mg/kg (Figure 1A).

### Electrically Induced Arterial Thrombosis in Rat Carotid Artery (*in vivo*)

(1) Dynamics of thrombus formation

**FIGURE 2:**
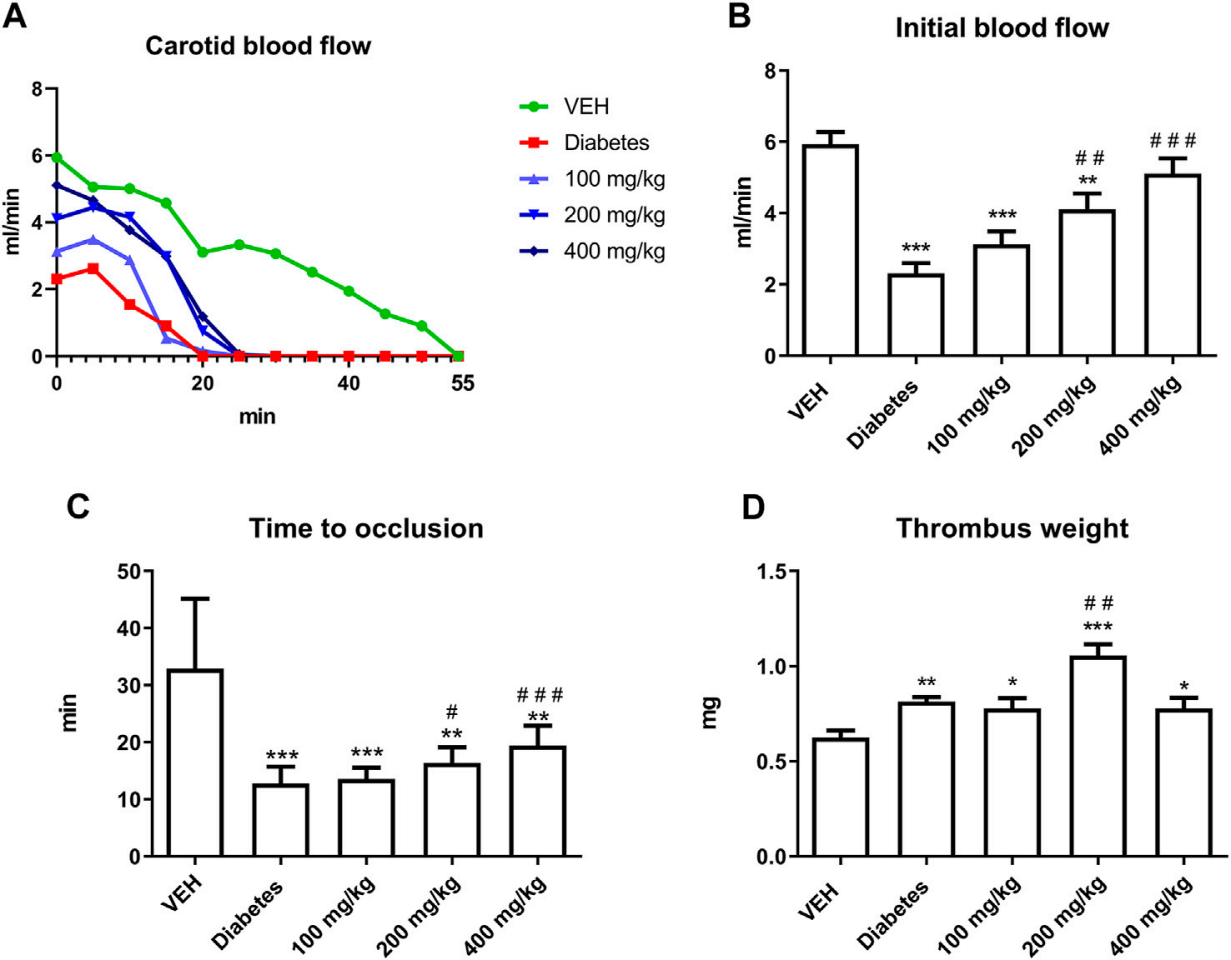
The effect of TE on electrically induced thrombosis. Changes in the carotid blood flow in the artery of rats subjected to electrical stimulation **(A)**. The effect of TE on: IBF in the rat artery before electrical stimulation **(B)**, TTO in the rat artery **(C)**, dry thrombus weight **(D)**. *p < 0.05, **p < 0.01, ***p < 0.001 vs. VEH; #*p* < 0.05, ##*p* < 0.01, ###*p* < 0.001 vs. Diabetes; *n* = 8–11. Data are shown as mean ± SEM.

TE increased the IBF and prolonged TTO at 200 and 400 mg/kg doses (Figures 2B,C). However, TE at 200 mg/kg dose increased thrombus weight (Figures 2D).

(1) Histological staining of the rat arterial thrombus

**FIGURE 3:**
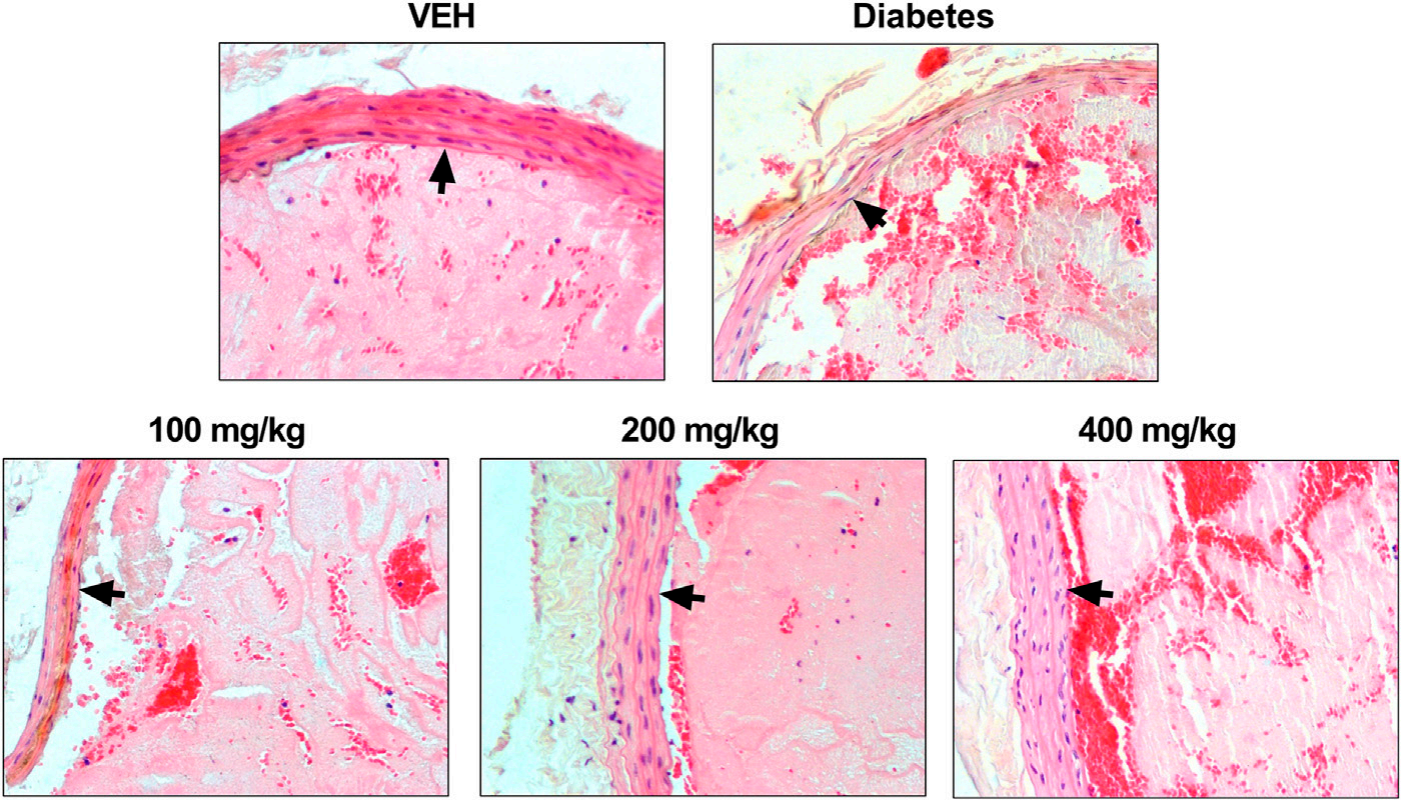
Representative photomicrographs of the thrombi and rat carotid artery wall. Fibrin and platelet aggregates are stained pink, erythrocytes are stained red, leukocytes are stained blue, and black arrows indicate the arterial wall. Routine H and E staining, ×200 magnification.

TE changed the arterial thrombus structure and arterial wall in animals from the test groups (Figure 3). All thrombi consisted of fibrin, platelets, trapped erythrocytes, and leukocytes in various proportions. Thinning of the middle layer of the arterial wall was observed in animals from the Diabetes group and in animals treated with TE at 100 mg/kg dose. The largest increase in the thickness of the middle layer of the arterial wall was observed in animals treated with TE at 400 mg/kg dose. The middle layer thickness in this group was similar to that observed in the VEH group. Furthermore, only single leukocytes were observed in thrombi from animals treated with TE at 400 mg/kg dose. The thrombi from the animals treated with TE at 200 mg/kg dose contained a large amount of fibrin. Many erythrocytes were entrapped in the thrombus in Diabetes animals and in animals treated with TE at 400 mg/kg dose.

### Thrombus Formation in the Flow Chamber and the Assessment of PI in Rat Blood (*ex vivo*)

**FIGURE 4:**
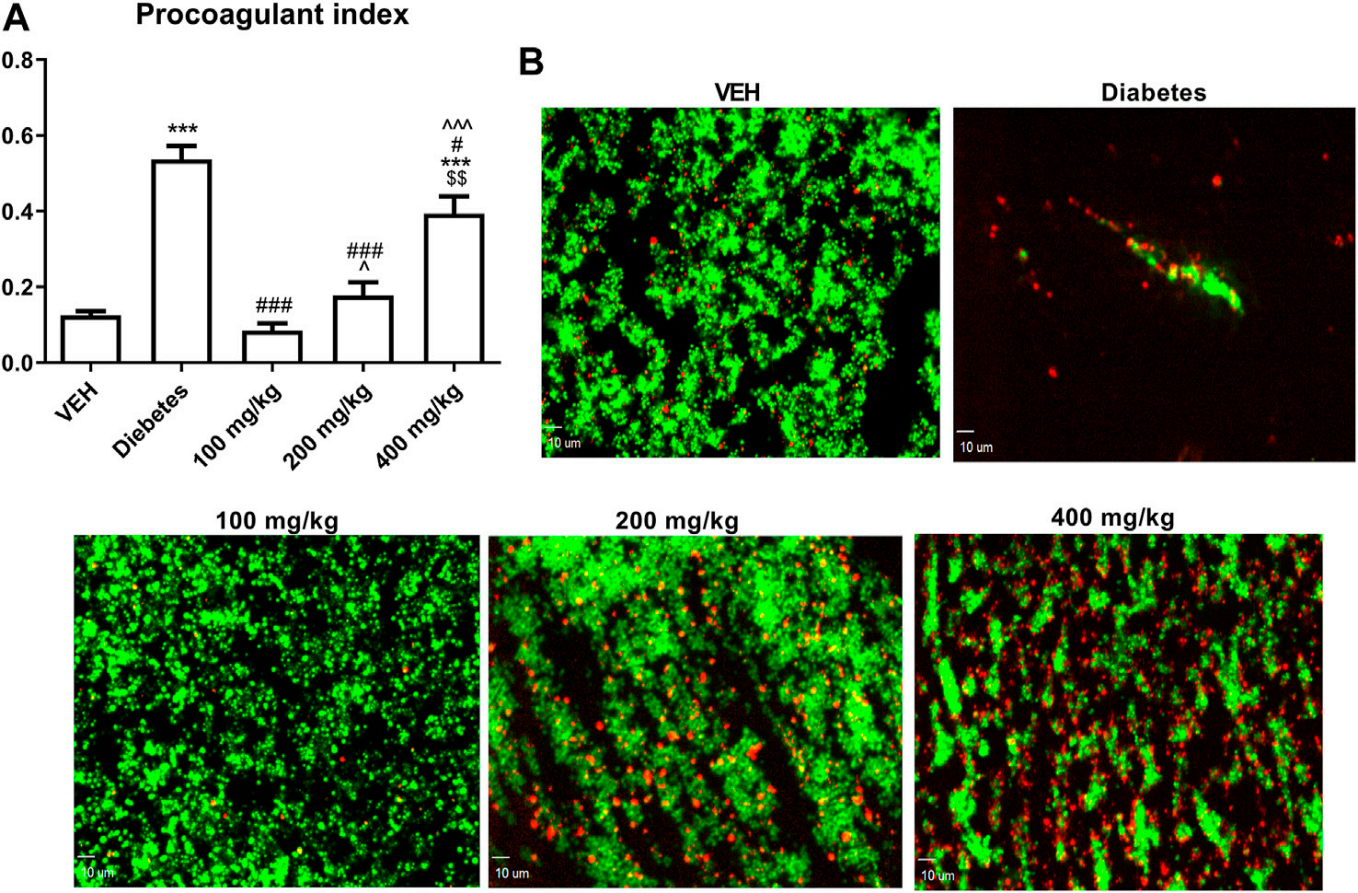
The effect of TE on PI **(A)**. Representative confocal microscopy images of thrombus consisting of PS-negative platelets (green) and PS-positive platelets (red). Bar = 10 µm **(B)** ***p < 0.001 vs. VEH; #*p* < 0.05, ###*p* < 0.001 vs. Diabetes; ^*p* < 0.05,^^^*p* < 0.001 vs. 100 mg/kg; $$*p* < 0.01 vs. 200 mg/kg; *n* = 8–11. Data are shown as mean ± SEM.

TE decreased PI at all the tested doses. However, this effect was less pronounced as the dose of TE increased (Figure 4A).

### Laser-Induced Thrombosis in the Mice Mesenteric Vein and the Assessment of the Thrombus Area and PECAM-1/Thrombus Ratio (*in vivo*)

**FIGURE 5:**
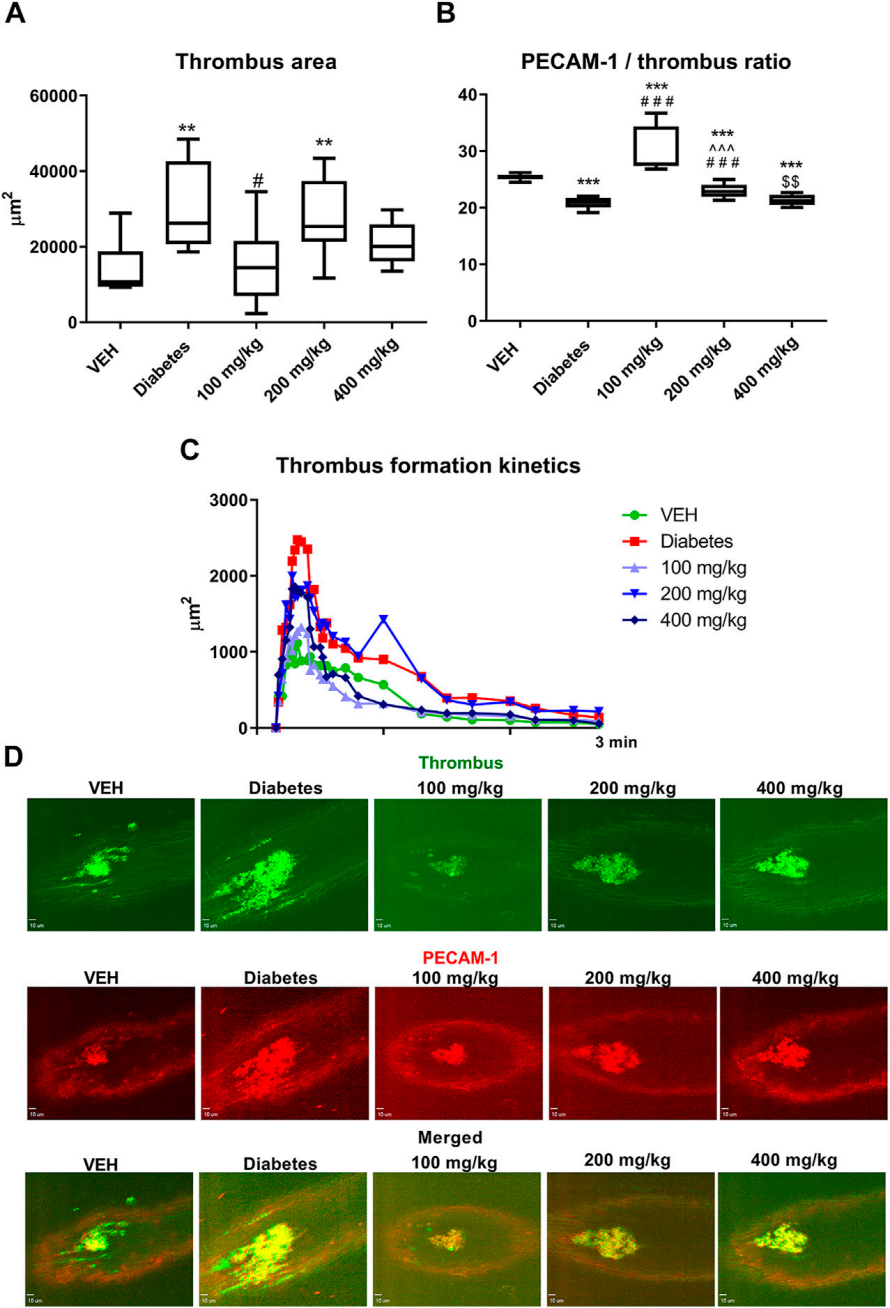
The effect of TE on laser-induced thrombosis. The effect of TE on: thrombus area **(A)** and PECAM-1/thrombus ratio **(B)**. Kinetics of thrombus formation at the site of laser injury **(C)**. Representative confocal microscopy images of thrombus (green, top row), PECAM-1 (red, middle row), and merged channels (bottom row). Bar = 10 µm **(D)**. **p < 0.01, ***p < 0.001 vs. VEH; #*p* < 0.05, ###*p* < 0.001 vs. Diabetes; ^^^*p* < 0.001 vs. 100 mg/kg; $$*p* < 0.01 vs. 200 mg/kg; *n* = 7–10. Data are shown as median (interquartile range).

TE at only 100 mg/kg dose decreased the thrombus area (Figure 5A). However, at 100 and 200 mg/kg doses, TE increased the PECAM-1/thrombus ratio, thus indicating reduced platelet activity (Figure 5B).

### Assessment of P-Selectin Secretion at the Site of Laser Injury in the Mice Mesenteric Vein (*in vivo*)

**FIGURE 6:**
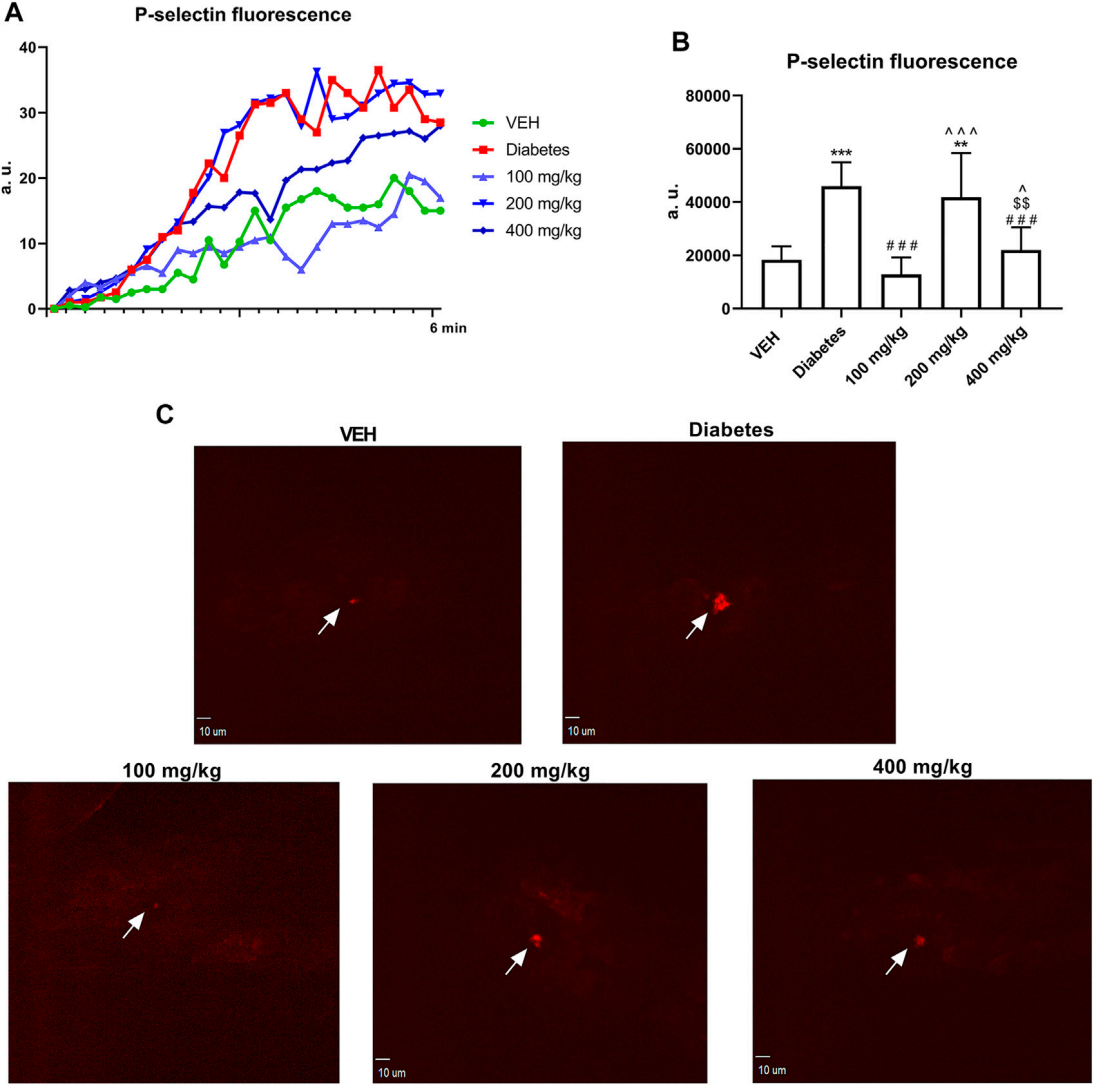
Changes in P-selectin fluorescence at the site of injury. Fluorescence of P-selectin is presented in arbitrary units of fluorescence (a. u.) **(A)**. The effect of TE on P-selectin secretion by platelets **(B)**. Representative confocal microscopy images of P-selectin. White arrows indicate the site of injury. Bar = 10 µm **(C)**. **p < 0.01, ***p < 0.001 vs. VEH; ###*p* < 0.001 vs. Diabetes; ^*p* < 0.05, ^^^*p* < 0.001 vs. 100 mg/kg; $$*p* < 0.01 vs. 200 mg/kg, *n* = 8–9. Data are shown as mean ± SEM.

TE at 100 and 400 mg/kg doses decreased P-selectin secretion at the site of laser injury of the mesenteric vein (Figure 6B).

### Evaluation of Fibrin Net Density in Rat Plasma (*ex vivo*)

**FIGURE 7:**
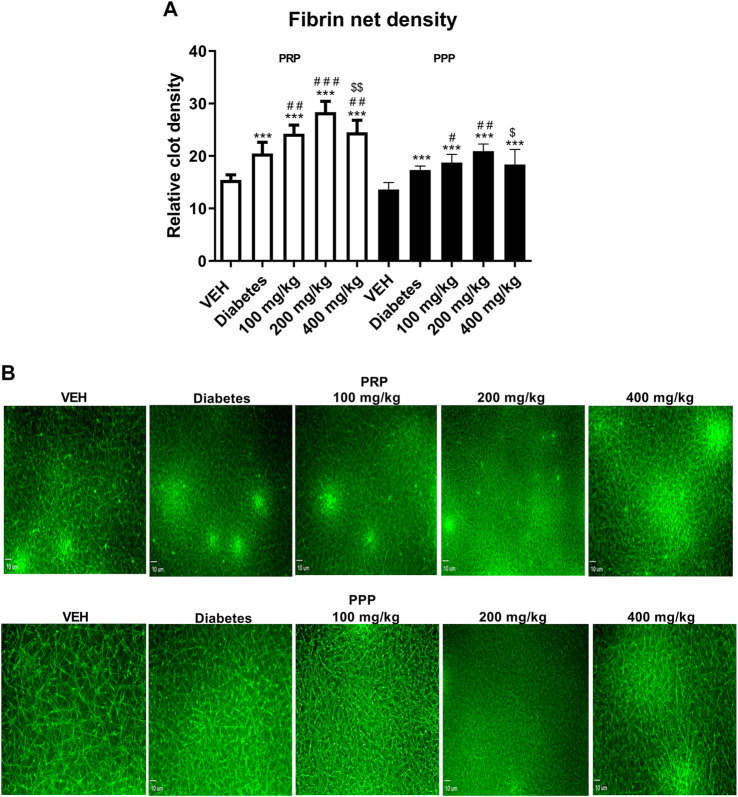
The effect of TE on the fibrin net density in clots formed after recalcination of PRP (white bars) and PPP (black bars) **(A)**. Representative confocal microscopy images of fibrin net. Bar = 10 µm **(B)**. ***p < 0.001 vs. VEH; #*p* < 0.05, ##*p* < 0.01, ###*p* < 0.001 vs. Diabetes; $*p* < 0.05, $$*p* < 0.01 vs. 200 mg/kg; *n* = 7–9. Data are shown as mean ± SEM.

Fibrin net density was assessed in the clot formed after recalcination of PRP and PPP. The use of two different experimental environments (PPP and PRP) enabled to assess the role of platelets in the process of fibrin formation after TE treatment. TE increased the relative clot density in PRP at all the tested doses, and the largest increase was observed at 200 mg/kg (Figure 7A, white bars). However, the increase in the relative clot density in PPP was observed only at 100 and 200 mg/kg doses (Figure 7A, black bars). Similar to PRP, the most pronounced increase in the relative clot density in PPP was observed at 200 mg/kg dose.

### TF Expression at the Site of Laser Injury in the Mesenteric Artery in Mice (*in vivo*)

**FIGURE 8:**
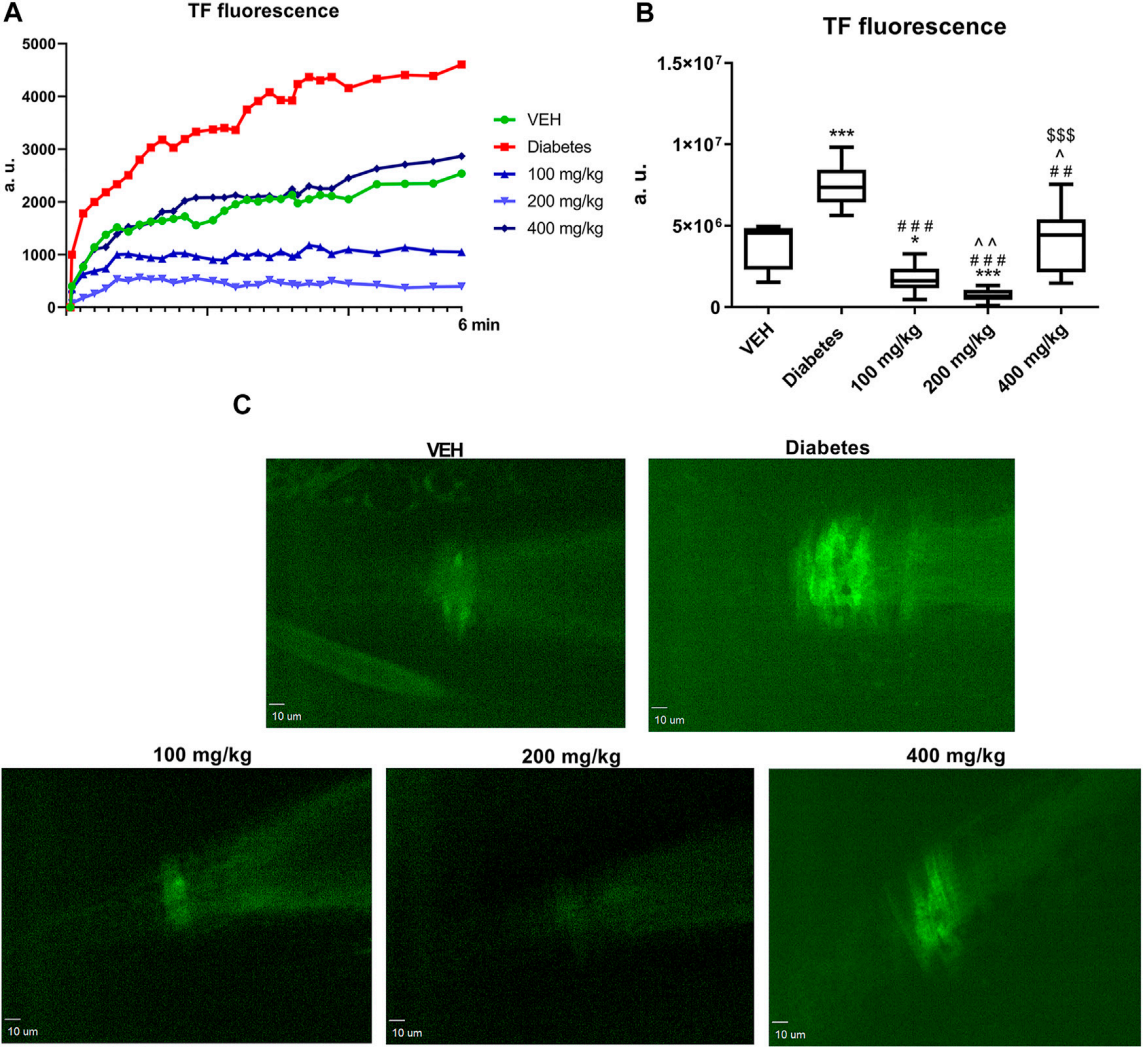
Changes in TF fluorescence at the site of injury over time. Fluorescence of TF is presented in arbitrary units of fluorescence (a. u.) **(A)**. The effect of TE on TF expression **(B)**. Representative confocal microscopy images of TF expression. Bar = 10 µm **(C)**. *p < 0.05, ***p < 0.001 vs. VEH; ##*p* < 0.01, ###*p* < 0.001 vs. Diabetes; ^*p* < 0.05, ^^*p* < 0.01 vs. 100 mg/kg; $$$*p* < 0.001 vs. 200 mg/kg; *n* = 7–9. Data are shown as median (interquartile range).

TE at all the tested doses decreased TF fluorescence at the site of laser injury in the mesentery artery. This effect was most pronounced at 200 mg/kg dose (Figure 8B).

### Fibrinolysis

**FIGURE 9:**
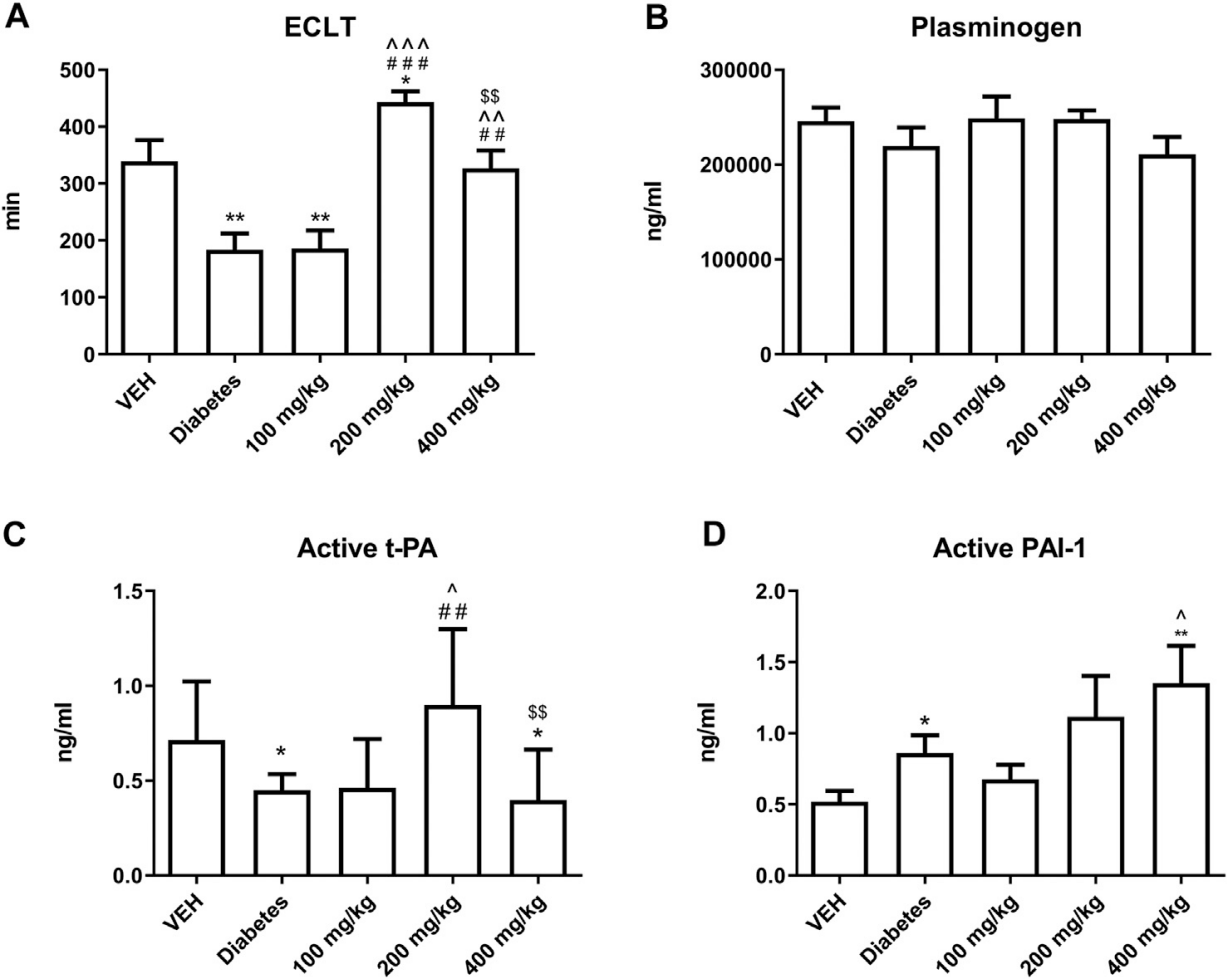
The effect of TE on fibrinolysis. The effect of TE on: ECLT **(A)**, plasminogen concentration **(B)**, concentration of active form of t-PA **(C)**, concentration of active form of PAI-1 **(D)**. *p < 0.05, **p < 0.01 vs. VEH; ##*p* < 0.01, ###*p* < 0.001 vs. Diabetes; ^*p* < 0.05, ^^*p* < 0.01, ^^^*p* < 0.001 vs. 100 mg/kg; $$*p* < 0.01 vs. 200 mg/kg; *n* = 7–11. Data are shown as mean ± SEM.

TE prolonged ECLT at 200 and 400 mg/kg doses. This effect was most prominent at 200 mg/kg dose (Figure 9A). TE did not affect the concentration of plasminogen (Figure 9B). TE at 200 mg/kg dose increased the concentration of active form of t-PA, but its concentration was reduced at 400 mg/kg dose as compared to that at 200 mg/kg dose (Figure 9C). TE showed a tendency to increase the concentration of active form of PAI-1 (Figure 9D).

### Concentrations of NO_2_^−^ and NO_3_^−^

TE at 200 mg/kg dose increased the concentrations of NO_2_^−^ and NO_3_^−^. However, TE at 400 mg/kg dose decreased the concentrations of NO_2_^−^ and NO_3_^−^ as compared to that at 200 mg/kg dose (Figure 1B).

### Concentrations of 6-Keto PGF_1α_ and TNF-α

TE at 400 mg/kg dose decreased the concentration of 6-keto PGF_1α_ (Figure 1C). However, it did not affect the concentration of TNF-α (Figure 1D).

## Discussion

In the present study, we observed the multidirectional effects of TE on hemostasis in STZ-diabetic rats and mice, which were not associated with the blood glucose level. An increase in the dynamics and extent of thrombus formation in STZ-diabetes was demonstrated in the models of electrically induced and laser-induced thrombosis. Thrombosis was enhanced due to increased platelet (Figures 4–6) and coagulation activity (Figures 7, 8), impaired fibrinolysis (Figures 9C,D), endothelial dysfunction (Figure 1B), reduced blood flow (Figure 2B), and inflammatory state (Figures 1C,D). *In vivo* experiments were performed in both rats and mice to make the overall effect of TE species-independent. We observed that TE in diabetic rats, unlike in normoglycemic rats (Marcinczyk et al., 2017), does not exert antithrombotic effect in the rat carotid artery. Moreover, it intensified electrically induced thrombotic process at 200 mg/kg dose, which was manifested as increased thrombus weight (Figure 2D). Considering the slight increase in fibrin formation in normoglycemia, we presumed that the increase in thrombus weight in STZ-diabetes could be due to enhanced coagulation. This hypothesis was confirmed in the experiment where the most pronounced fibrin net density was observed at TE dose of 200 mg/kg in clots formed in both PPP and PRP. TE at all the tested doses increased fibrin net density in PRP, while it increased the fibrin net density in PPP only at 100 and 200 mg/kg doses. This observation indicated an increase in platelet activity at 400 mg/kg dose as compared to that noted at 100 mg/kg dose and confirmed the key role of platelets in the process of fibrin formation. Increased fibrin net density was the reason for further studies on the effect of TE on coagulation. Because the TF/VIIa complex triggers coagulation *in vivo* (Smith, 2009), the next stage of the study was to determine the expression of TF in a mouse mesenteric artery. TE at all the tested doses decreased the expression of TF, and this effect was the strongest at 200 mg/kg dose (Figure 8). Because TE at 200 mg/kg dose caused the most dense fibrin net formation, the contribution of TF in the enhancement of coagulation was ruled out. As platelets play an essential role in the process of fibrin formation, the next stage of the study was to evaluate the influence of TE on platelet procoagulant activity. For this purpose, we used a flow chamber combined with a confocal microscopic imaging system. This model enables real-time monitoring of the process of thrombus formation on collagen fibers (*ex vivo*). Platelets in thrombus can be divided into two subpopulations: procoagulant and aggregating platelets. During sustained and potent activation, platelets change their discoidal shape to irregular shape, and the platelet cell membrane undergoes irreversible reorganization, which causes exposure of PS from the inner to the outer leaflet of the platelet plasma membrane. Because PS catalyzes the activation of coagulation factors, platelets with exposed PS are called procoagulant platelets. Aggregating platelets do not show exposure of PS (PS-negative platelets). However, because they are close to activating factors, they form pseudopods and undergo secretion (Heemskerk et al., 2013). As PS catalyzes coagulation reactions, the ratio of the area of PS-positive platelets to the area of PS-negative platelets is termed as PI and reflects the extent of platelet procoagulant response. PI was reduced at all tested doses of TE; the strongest antiprocoagulant effect was observed at 100 mg/kg dose, while the weakest reduction in PI was observed at 400 mg/kg dose (Figure 4). Therefore, it can be assumed that the weakened antiprocoagulant effect contributed to the increased fibrin net density in clots formed in PRP at 400 mg/kg dose compared to that in the Diabetes group. However, the decreased fibrin net density at 400 mg/kg dose as compared to that at 200 mg/kg dose with and without platelets (PRP and PPP) indicates an unknown plasma-related mechanism of coagulation activation. Further experiments on the influence of TE on platelet activity were performed in a model of laser-induced thrombosis. In this model, because of the small area of the exposed subendothelial matrix, potent and sustained platelet activation does not occur, which results in thrombus composed of platelets that do not undergo irreversible activation (aggregating, PS-negative platelets) and can easily detach from the site of injury. Therefore, this model is suitable to investigate platelet activity in the aggregation state. TE only at the lowest dose (100 mg/kg) decreased the thrombus area in the model of laser-induced thrombosis (Figure 5A); this finding is consistent with the antiplatelet activity observed in the flow chamber model. The previously observed antithrombotic effect in normoglycemic rats and mice was noted only at the highest dose (400 mg/kg) and corresponded to a potent antiplatelet activity (Marcinczyk et al., 2017). Next, the assessment of the activity of aggregating platelets in thrombus was measured by the PECAM-1/thrombus ratio, which indicates the proportion of platelet-endothelial cell adhesion molecule 1 (PECAM-1) in thrombus. As PECAM-1 is considered as an antithrombotic molecule, the higher the PECAM-1/thrombus ratio, the less activated are the platelets in thrombus (Marcinczyk et al., 2020). TE increased the PECAM-1/thrombus ratio with the most pronounced effect at 100 mg/kg dose (Figure 5B). The antiplatelet effect of TE at 100 mg/kg dose was significant enough to translate into the antithrombotic effect. These observations indicate that TE at 100 mg/kg dose most effectively reduced platelet activity in two modes of activation: reversible, which was expressed as increased PECAM-1/thrombus ratio, and irreversible, which was expressed as decreased PI. The sections of arterial thrombi showed an increase in entrapped erythrocytes at 400 mg/kg dose (Figure 3). This indicates hemolysis, which leads to the enhanced incorporation of erythrocytes into thrombus (Helms et al., 2013). Furthermore, the pale red color of plasma of the 400 mg/kg group (data not shown) indicated the occurrence of hemolysis. During hemolysis, erythrocytes release platelet agonists, (e.g., ADP), which also contributes to enhanced platelet activity (Noh et al., 2010) and could partially explain the alleviation of the antiplatelet effect of TE at 400 mg/kg dose. The next stage of the study was the assessment of P-selectin secretion at the site of laser-induced mesenteric vein injury under intravital conditions. P-selectin, a marker of platelet secretion, is responsible for thrombus progression and its stability, which allows direct interactions of platelets with endothelial cells and leukocytes (Prakash et al., 2017). Inhibition of P-selectin secretion was observed only at 100 and 400 mg/kg doses; this finding may suggest the decreased stability of thrombus (Figure 6). The curve of thrombus formation kinetics in the 100 mg/kg- and 400 mg/kg-treated groups is characterized with a high peak and the slope of the curve, which correspond to the addition of the thrombotic material shortly after vessel injury and subsequent elution of the thrombus. The curve of thrombus kinetics in the 200 mg/kg-treated group shows two peaks that correspond to two massive additions of the thrombotic material; this finding suggests increased thrombus stability as compared to that noted in the 100 and 400 mg/kg groups (Figure 5C). We observed that TE at 200 and 400 mg/kg doses increased the IBF in the rat carotid artery and prolonged TTO (Figures 2B,C); this finding indicated the contribution of the vessel wall in changes of thrombus formation kinetics. Furthermore, TE at 400 mg/kg dose also prolonged BT (Figure 1A), which reflects the dependence of primary hemostasis on the blood vessel response (vasoconstriction/vasodilatation) and on the activity of platelets that form a platelet plug at the site of injury (Mattix and Singh, 1999). Because of the lack of the antiplatelet effect of TE at 400 mg/kg dose in this model, the prolongation of BT indicated an improvement in vascular function. Furthermore, H and E staining revealed that TE increased the thickness of the middle layer of the artery wall (Figure 3), which may be an indirect effect associated with an increased blood flow (Basu et al., 2010). Some of the compounds of TE and their metabolites have potential to improve vascular functions, which may be reflected as increased blood flow observed in Figure 2B. Metabolites generated from ellagic acid and ellagitannins by the intestinal microbiota such as urolithin A and B have been shown to enhance eNOS expression (Han et al., 2016; Spigoni et al., 2016), and the activity of procyanidin B leads to vasorelaxation of the human mammary artery (Novakovic et al., 2017). This indicated that the observed improvement in blood flow might be partially due to an endothelial-dependent mechanism. Therefore, in the next stage of the study, the concentrations of NO_2_^−^ and NO_3_^−^, the stable metabolites of NO – the main vasoactive molecule released from the endothelium (Mitchell et al., 2008) – were measured. TE at 200 mg/kg dose increased the concentrations of NO_2_^−^ and NO_3_^−^, which may contribute to IBF improvement and TTO prolongation (Figures 2B,C, respectively). Previous studies on endothelial NO release have shown that the increase in eNOS activity is linked to the decrease in TF expression (Solovey et al., 2010). Therefore, the largest decrease in TF fluorescence at 200 mg/kg dose may be due to the increased production of NO at this dose. However, the reason why NO_2_^−^ and NO_3_^−^ concentrations increased only at 200 mg/kg dose and not at 400 mg/kg dose remains unclear.

The next component of the prothrombotic effect of TE at 200 mg/kg dose was the attenuation of fibrinolysis, as the fibrinolytic activity of plasma measured as ECLT was most strongly inhibited at this dose (Figure 9A). However, TE at 200 mg/kg dose increased the concentration of the active form of t-PA (Figure 9C), which might be due to increase NO release (Giannarelli et al., 2007). Furthermore, compared to 200 mg/kg dose, TE at 400 mg/kg dose decreased the concentration of active form of t-PA, which may result from decreased concentration of bradykinin – one of the activators of t-PA release (Marcinczyk et al., 2017). The antifibrinolytic effect of TE could also be, to some extent, explained by an increase in the concentration of the active form of PAI-1 (Figure 9D). This may be due to the increased activation of platelets, which release PAI-1 during the activation process (Morrow et al., 2020). Furthermore, the tendency of increasing concentration of active form of PAI-1 may be due to the decreased concentration of t-PA that binds to PAI-1, thus diminishing the measurable pool of free PAI-1 (Chandler et al., 1995).

Considering the anti-inflammatory activity of TE (Tunón et al., 1995; Wölfle et al., 2017) and the prothrombotic effect of inflammation (Esmon, 2003), the concentration of TNF-α, a commonly accepted inflammation marker, was measured. TE did not change the level of TNF-α in rat plasma (Figure 1D). However, no effect on TNF-α does not imply a lack of anti-inflammatory properties of TE in diabetes. The less number of leukocytes in thrombi from the 400 mg/kg-treated group than that in thrombi from the Diabetes group may indicate the anti-inflammatory activity of TE (Figure 3). We have also shown that TE at 400 mg/kg dose lowered the concentration of 6-keto PGF_1α_, thus indicating the inhibition of PGI_2_ production by endothelial cells in the presence of high dose of TE (Figure 1C). The effect of decreased PGI_2_ production may be due to the known inhibitory effect of TE or its agrimoniin-rich fraction on COX activity (Tunón et al., 1995; Hoffmann et al., 2016). Furthermore, the results of our study are consistent with those which show that the anti-inflammatory effect of TE is dependent on the inhibition of arachidonic acid (AA) metabolism. However, in those studies, the anti-inflammatory effect was demonstrated in a human keratinocyte cell line (HaCaT, *in vitro*) and in human skin (*in vivo*, applied topically) (Hoffmann et al., 2016). Thus, our study for the first time showed that after oral intake, the anti-inflammatory effect of TE could be dependent on the inhibition of AA metabolism.

In our present study, the bioavailability and plasma concentrations of specific compounds originating from TE and their metabolites were not assessed; thus, we cannot confirm which compounds or metabolites are primarily responsible for the changes observed in hemostasis. Furthermore, it is possible that the inflammatory state changed the bioavailability of specific TE components. Urolithins, which are the gut microbiota-derived metabolites of ellagitannins, undergo glucuronidation rapidly after absorption. Glucuronides of urolithins are inactive, but under inflammatory conditions, they are deconjugated by ß-glucuronidase to free urolithins (Piwowarski et al., 2017). Therefore, it can be presumed that the amount of free urolithins after the administration of equivalent doses of TE is different in normoglycemia and STZ-diabetes. Furthermore, the differences in gut microbiota in normoglycemia and diabetes (Ma et al., 2020) could lead to the formation of different metabolites of TE. In our present study, we showed for the first time that the activity of TE is dependent on the pathological condition. It is also possible that TE activity in STZ-diabetes was associated with the production of inflammatory-related TE metabolites. To summarize, we have shown that TE exerts multidirectional effects on the activity of platelets, coagulation, fibrinolysis, and endothelial-dependent vascular functions in STZ-diabetic rats and mice. These multidirectional effects of TE on hemostasis translated into a model-dependent effect on the thrombotic process. Furthermore, the contrasting effects of TE in normoglycemia (Marcinczyk et al., 2017) and diabetes indicates that the mechanism of action of TE is related to the pathological-dependent baseline hemostatic activity.

Since the TE has been used to treat diarrhea (Tomczyk and Latté, 2009), our study may have clinical relevance in hemostasis regulation. However, considering the activation of coagulation and the inhibition of fibrinolysis by TE, the beneficial impact of ellagitannins (Haber et al., 2011; Istas et al., 2018) on hemostasis after short-term use is not so obvious. Our study’s TE’s doses were selected based on studies with rats and mice (Shushunov et al., 2009). Pharmacokinetics of TE in humans have not been investigated yet, but TE’s doses are lower (approximately 2.5–40 mg/kg) (Subbotina et al., 2003; Huber et al., 2007). Due to the fast metabolism rate, mice and rats often require higher doses of the drug (e.g., acetylsalicylic acid) (Wientjes and Levy, 1988; Nagelschmitz et al., 2014). Nevertheless, we cannot state whether the same pharmacological effects can be expected after using the same doses of TE in humans as in rodents. Raising these issues, further studies are needed to assess the potential clinical significance of our study particularly in patients with increased risk of thromboembolic events.

## Data Availability

The raw data supporting the conclusion of this article will be made available by the authors, without undue reservation.

## Abbreviation

6-keto PGF_1α_, 6-keto prostaglandin 1 α; AA, arachidonic acid; ANX V, annexin V; DiOC6(3), 3,3ʹ-dihexyloxacarbocyanine iodide; ECLT, euglobulin clot lysis time; IBF, initial blood flow; LC-ESI-MS, liquid chromatography-electrospray ionization-mass spectrometry; NO_2_^−^ and NO_3_^−^, nitrite and nitrate; PAI-1, plasminogen activator inhibitor 1; PGI_2_, prostacyclin; PI, platelet procoagulant index; PPP, platelet-poor plasma; PRP, platelet-rich plasma; PS, phosphatidylserine; *i.p.*, intraperitoneal administration (*lat. iniectio intraperitonealis*); *p.o.*, oral administration (*lat. per os*); TE, tormentil extract; TF, tissue factor; TNF-α, tumor necrosis factor α; t-PA, tissue plasminogen activator; TTO, total time to occlusion; TXA_2_, thromboxane; STZ, streptozotocin.
